# First report of *Meloidogyne javanica* (Nematoda: Meloidogynidae) infecting *Scoparia dulcis*, a medicinal plant in Brazil

**DOI:** 10.21307/jofnem-2019-072

**Published:** 2019-10-14

**Authors:** Cristiano Bellé, Rodrigo Ferraz Ramos, Andressa Lima de Brida, Tiago Edu Kaspary

**Affiliations:** 1 Departamento de Solos, Universidade Federal de Santa Maria, Santa Maria, Brazil; 2 Instituto de Biologia, Universidade Federal de Pelotas, Pelotas, Brazil; 3 Instituto Nacional de Investigación Agropecuaria-INIA La Estanzuela, Colonia, Uruguay

**Keywords:** Detection, Diagnosis, Identification, Root-knot nematodes, Sweet broom

## Abstract

Medicinal plants *Scoparia dulcis* showing symptoms caused by root-knot nematodes were detected in the municipality of Cachoeira do Sul, Rio Grande do Sul State, Brazil. Based on the morphological, esterase phenotypes, and molecular analyses of the mitochondrial DNA region between the cytochome oxidase subunit II and 16S rRNA genes (mtDNA) and species-specific sequence characterized amplified region (SCAR), the causal agent of the observed symptoms was identified as *Meloidogyne javanica*. Pathogenicity was confirmed by fulfilling a modified version of Koch’s postulates. To our knowledge, this is the first report of *M. javanica* infecting *S. dulcis* in Brazil.

*Scoparia dulcis* L. (Scrophulariaceae), commonly known as sweet broom, is a perennial plant, widely distributed in tropical and subtropical regions, especially in flooded environments (Jain and Singh, 1989; Zhang et al., 2014). *Scoparia dulcis* is an erect, annual herb with serrated leaves, producing white flowers. Its ethnomedicinal uses among various indigenous tribes in the rain-forest is well-documented (Murtl et al., 2012). Pharmacological properties of *S. dulcis* have been speculated and traditionally used as a treatment for diabetes mellitus, hypertension, stomach troubles, hypertension, inflammation, bronchitis, hemorrhoids, hepatosis, and as an analgesic and antipyretic (Das and Chakraborty, 2011; Murtl et al., 2012).

Medicinal plants can be attacked by pests, diseases, and plant-parasitic nematodes, which can qualitatively and quantitatively impair curative properties and production (Bellé et al., 2017; Pandey, 2017). Among the plant-parasitic nematodes, the most important genus is *Meloidogyne* Göldi, 1887, which, cause to direct damage such as root galls and reduction in the number of roots, and predisposition to fungal and bacterial diseases, causing losses in crop yields (Karssen, 2002; Rich et al., 2008).

Sweet broom plants showing distinctive root galls (Fig. 1A) were collected on August 2018 in the municipality of Cachoeira do Sul, Rio Grande do Sul State, Brazil. In order to identify the plant-parasitic nematode species, a combination of morphological, biochemical, and molecular analyses were performed.

**Figure 1:**
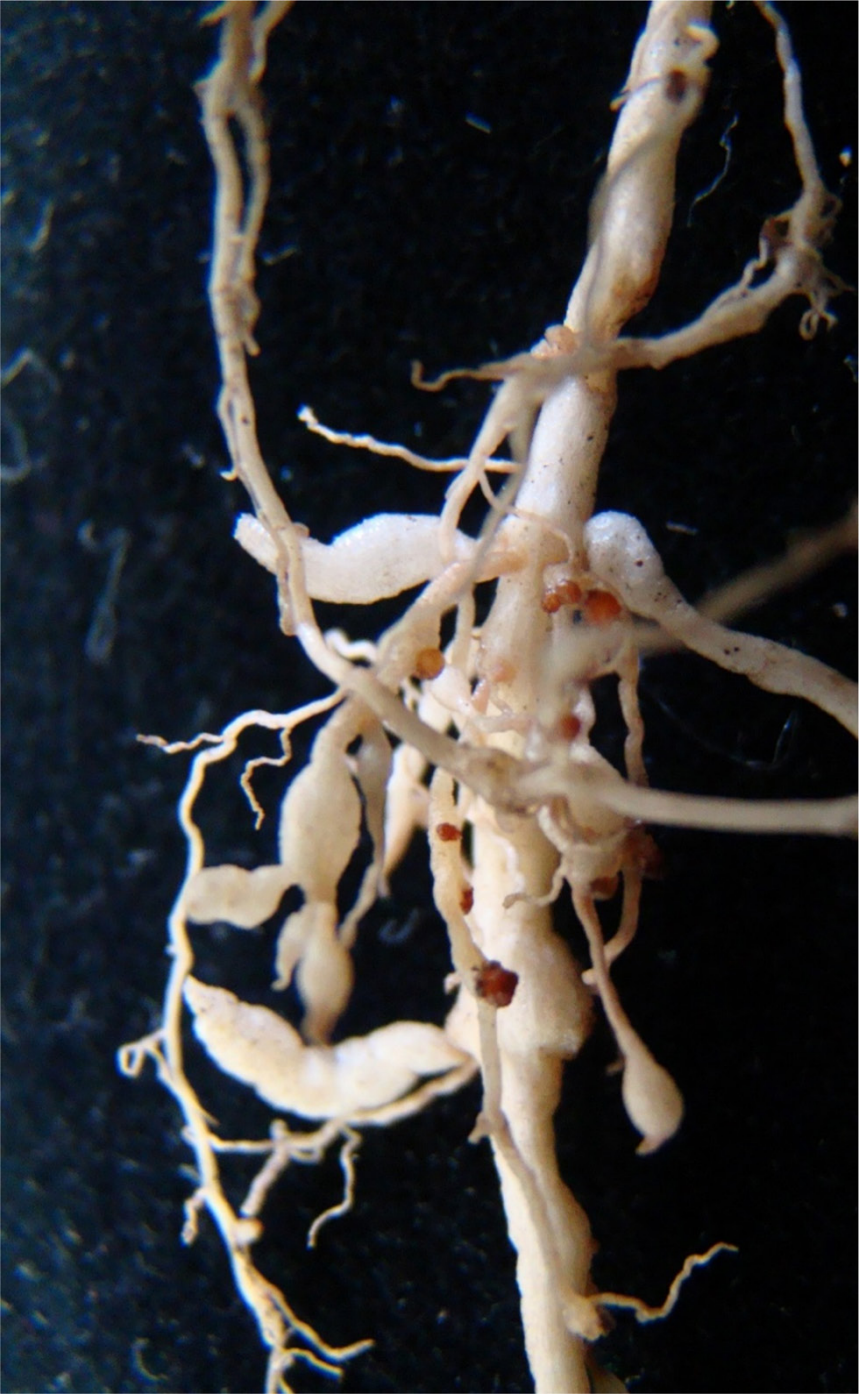
Sweet broom (*Scoparia dulcis* L.) roots showing galls caused by *Meloidogyne javanica* (Treub, 1885) Chitwood, 1949 infection.

To identify this *Meloidogyne* population, the following techniques were used: esterase phenotypes (*n* = 40 females) (Carneiro and Almeida, 2001); morphology and morphometrics of second-stage juveniles (J2) (*n* = 40) and females (*n* = 20), and perineal patterns (*n* = 20); and molecular characterization of the mitochondrial DNA region between the cytochome oxidase subunit II (COII) and 16S rRNA genes (mtDNA) using the primers C2F3 (5´-GGTCAATGTTCAGAAATTTGTGG-3´) and 1108 (5´-TACCTTTGACCAATCACGCT-3´) (Powers and Harris, 1993) along with PCR species-specific sequence characterized amplified region (SCAR) for confirmation, using a primer set composed of Fjav (5´-GGTGCGCGATTGAACTGAGC-3´) and Rjav (5´-CAGGCCCTTCAGTGGAACTATAC-3´) (Zijlstra et al., 2000).

The nematode population density observed in the sample was 1,976 J2s/g of roots. The morphometric study showed the following results; J2s: length (L) = 414.5 ± 36.1 (345.0–470.5) µm, a = 33.7 ± 2.8 (28.1–32.5), c = 8.4 ± 0.9 (6.3–10.1), stylet length = 11.5 ± 1.2 (10.1–14.2) µm, dorsal esophageal gland orifice (DGO)  = 3.5 ±  0.4 (3.0–4.6) µm, tail length = 51.1 ± 3.3 (45.8–54.4) µm and hyaline tail terminus = 11.5 ± 1.7 (9.1–14.7) µm; and females L = 850.5 ± 90.5 (640.5–995.5) µm, stylet length = 14.5 ± 0.8 (12.5–15.8) µm, and DGO = 3.4 ± 0.7 (2.8–4.2) µm. The perineal patterns showed a low dorsal arch, trapezoid shape, smooth striae interrupted by a pair of incisures on both sides corresponding to lateral fields, clearly demarcated from striae by parallel lines, tail whorl often distinct. The overall morphology and morphometrics of the population of *M. javanica* (Treub, 1885) Chitwood, 1949 are in agreement with the original description (Rammah and Hirschmann, 1990; Hunt and Handoo, 2009).

The polymorphisms of the esterase bands by electrophoresis revealed the phenotype J3 (Rm = 1.00, 1.25, and 1.40) typical of *M. javanica* (Carneiro et al., 1996). The mtDNA sequence (1639 bp) was submitted to GenBank with Accession No. MN227142. Searches on BLAST showed that 99% identity with sequences of *M. javanica* isolates from South Africa (GenBank JX987326), Taiwan (GenBank JX100439), Turkey (GenBank LT602894), and Vietnam (GenBank MK033441). The PCR amplification using SCAR technique produced a specific fragment of expected size (∼670 bp) for *M. javanica* (Zijlstra et al., 2000).

In greenhouse tests, *S. dulcis* plantlets were maintained in pots with 2,000 dm^3^ sterilized soil. In total, 10 replicates were inoculated with 5,000 eggs and J2s from the original population of *M. javanica*, in addition to a non-inoculated control. Plants were well maintained under greenhouse conditions at 25 to 27°C. After 55 days, the inoculated plants exhibited galled root systems similar to plants observed in the field, with a nematode reproduction factor (final population/initial population) of 24.5. The non-inoculated plants did not exhibit any galls. The morphological and molecular characterization of this re-isolated root-knot nematode were identical those of *M. javanica*.

This is the first report of *M. javanica* parasitising *S. dulcis* in Brazil. Globally, *M. javanica* is considered an economically important agricultural nematode reported over 770 plant species, including tea [(*Camellia sinensis* (L.) Kuntze], grape (*Vitis* sp. L.), many vegetables, fruit trees, weeds, cereals, and ornamentals, causing severe yield losses (CABI, 2019). Hence, further studies are necessary to investigate the damage it can causes to specific crops, such as *S. dulcis*, grown in tropical, subtropical, and temperate regions.
